# Exacerbation of central serous chorioretinopathy during trauma-confronting psychotherapy– a case report

**DOI:** 10.1186/s12888-024-05756-6

**Published:** 2024-05-16

**Authors:** Eva Schäflein, Christian Mardin, Eva Morawa, Sophia Rudolf, Yesim Erim, Cosima Rhein

**Affiliations:** 1 Department of Psychosomatic Medicine and Psychotherapy, University Hospital of Erlangen, Friedrich- Alexander University Erlangen-Nürnberg (FAU), Erlangen, Germany; 2 Department of Psychotherapy and Psychosomatic Medicine, Faculty of Medicine, Technische Universität Dresden, Dresden, Germany; 3 Department of Ophthalmology, University Hospital of Erlangen, Friedrich-Alexander University Erlangen- Nürnberg (FAU), Erlangen, Germany

**Keywords:** Central serous chorioretinopathy, Post-traumatic stress disorder, Trauma-focused psychotherapy, Eye movement desensitization and reprocessing, Case report

## Abstract

**Background:**

Psychotherapy for post-traumatic stress disorder, in particular trauma-confronting psychotherapy, can be associated with increased stress. However, research on the somatic impact and psychosomatic interactions of these psychological stress reactions is lacking. We report on a 43-year old man whose central serous chorioretinopathy exacerbated upon trauma-confronting psychotherapy.

**Case presentation:**

We report on a man with pre-diagnosed, asymptomatic central serous chorioretinopathy who underwent inpatient psychosomatic therapy. He disclosed a history of sexual abuse by a family member and consequently showed intrusions, flashbacks, nightmares, avoidance behavior, and hyperarousal. Thus, we diagnosed post-traumatic stress disorder. After a stabilization phase, he underwent trauma-focused psychotherapy including trauma confrontation. In the course of this treatment, acute vision loss with blurred vision and image distortion of his right eye occurred. An ophthalmologic visit confirmed a relapse of a pre-diagnosed central serous chorioretinopathy. The analysis of stress biomarkers showed a decrease in testosterone levels and a noon peak in diurnal cortisol secretion, which is indicative of a stress reaction.

**Conclusion:**

Central serous chorioretinopathy may exacerbate upon psychotherapeutic treatment. In this case, an exacerbation of chorioretinopathy was observed in direct relation to the therapeutic intervention. Psychotherapists and ophthalmologists should collaborate in the psychotherapeutic treatment of patients with chorioretinopathy. Our case demonstrates the need to consider the possible increased stress levels during psychotherapy and resulting physical side effects, such as exacerbation of an existing condition. It is advisable to adjust the level of generated stress particularly well in the presence of stress-inducible physical diseases. Our case is a good example of the interplay between psychological and physical stress.

## Background

Central serous chorioretinopathy (CSC) is an eye disorder affecting mainly males with a mean age of 40 years. In its typical appearance, it is characterized by a thick choroid, a serous retinal detachment involving the area of the macula, and retinal pigment epithelium detachment. CSC appears to have a multifactorial pathogenesis, in which venous congestion, inflammation, ischemia, and changes in the hormonal and biochemical milieu seem to play a role [1]. In particular, increased levels of endogenous or exogenous glucocorticoids are associated with the development of CSC [2, 3]. Some studies have shown that the pathogenesis of CSC is strongly related with a dysregulation of the HPA axis, which plays an important role in the stress response and various mental disorders [4]. Accordingly, psychological stress is one of the most important risk factors [5]. A study of war veterans showed that post-traumatic stress disorder (PTSD) was strongly associated with the development of CSC [6].

One central component of PTSD treatment is trauma-confronting/ exposure psychotherapy, which leads to a temporary increase of stress [7]. Stress again can cause exacerbations of CSC [5]. Consequently, in patients suffering from PTSD with a history of CSC, stress-inducing trauma-oriented psychotherapeutic treatment including trauma exposure might have an impact on CSC. However, to our knowledge, there is no research reporting on the course of CSC in PTSD patients during a trauma-focused psychotherapeutic treatment.

## Case presentation

### Patient information

The 43-year old industrial mechanic had been married for three years and coupled with his wife for 16 years in total. He described the relationship as harmonic and supportive. The couple had no children and lived in a rental apartment. The patient had not been working for 1.5 years due to his psychiatric illness. Before that, high workload and limited capacities to perceive own boundaries regarding work and altruism for friends and family members had led to constant excessive demand. He received money from an occupational disability insurance and had applied for a disability pension (procedure ongoing). He did not have any debts or financial problems. He suffered also from a recurrent central serous retinopathy (CSR) which was stable at the timepoint of admission.

At admission, the patient reported intrusions, flashbacks, nightmares, avoidance behavior, and hyperarousal, in addition to sleep disturbances, tremor, and concentration problems. He described depressed mood, low energy, loss of interest, feelings of guilt and insufficiency, and rumination. Additionally, he reported exacerbations of arterial hypertension upon stressful situations.

The patient had received 50 sessions of ambulatory psychotherapy for depression six years before admission. At that time, the patient had not yet been diagnosed with PTSD due to considerable avoidance behavior. One year before admission, he had undergone a psychosomatic rehabilitation inpatient treatment. In the course of this treatment, memories of traumatic events had come up. During the three months before admission, he had received ambulatory psychotherapy. In addition, he received regular ambulatory psychiatric visits. These therapies had led to a temporary relief of depressive symptoms, which recurrently relapsed after a while. As it had been challenging to diagnose PTSD before due to lacking access to traumatic memories and then due to avoidance behavior, the patient had only been diagnosed with PTSD shortly before being recovered in our department.

The patient and his elder brother had grown up in an atmosphere of emotional neglect. Between the age of 10 and 13 years, the patient had been sexually abused by a family member and had no possibility to disclose his experiences to any family member due to a lack of trust. A distantly related family member had given him support and safety.

### Clinical findings

#### Psychopathological assessment

Patient awake, fully oriented, friendly. Subjective lack of concentration and retentiveness, which cannot be objectified during the visit. Depressed mood, anxious, feelings of guilt and insufficiency. Limited affective vibratory ability, reduced energy. Rumination. No signs of psychosis. No compulsions. Depersonalization. No relevant further dissociative symptoms. Intrusions, flashbacks, avoidance behavior, hyperarousal. Social withdrawal. No self-harming behavior. No signs of suicidality.

#### Psychometric assessment at admission

The Essen Trauma Inventory (ETI) [8] supported the diagnosis of a post-traumatic stress disorder (46 points). The Beck Depression Inventory (BDI-II) [9] suggested severe depressive symptoms in self-report (47 points). When compared to the clinician-rated diagnosis of a medium depressive episode, the higher score in self-report might have been associated with the patient’s feelings of insufficiency.

#### Physical examination findings

No noticeable problems apart from adiposity grade I WHO and a bursitis of the right shoulder.

#### Medication at inpatient admission

Sertraline 150 mg 1-0-0, quetiapine retard 25 mg 0-0-1, lercanidipine 10 mg 1-0-1, *metamizole 500 mg 1-0-1, ibuprofene 600 mg 1-0-1 (italics: was stopped in the course of the inpatient treatment)*.

##### Note:

The patient was not receiving corticosteroid therapy, either systemically or topically, which could impact the course of the disease.

#### Consultation of the department of ophthalmology (week 9 of inpatient stay)

On appearance, the patient reported reduced visual acuity on his right eye and suspected a recurrence of a known central serous retinopathy. Visual acuity was 20/50. The patient complained about blurred vision and micropsia. Clinical examination revealed a detachment of the central retinal pigment epithelium (RPE) and serous detachment of the central retina without hemorrhage or sign of exsudation on the right eye. OCT revealed a central elevation of neurosensory retina by 600 μm with an underlying detachment of RPE. In the course of follow-up, subretinal fluid was reduced substantially and RPE detachment decreased in height. As with OCT and by the clinical picture, macular neovascularization was ruled out, i.e. acute CSC recurrency was the diagnosis. His history showed already recurring episodes of CSC and there was no further report of other eye diseases or refractive error.

**Fig. 1:**
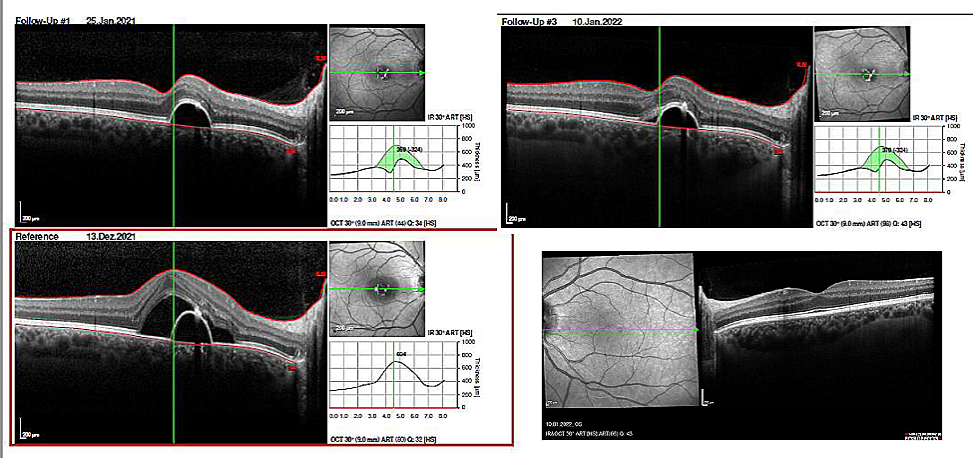
SD-OCT shows on the right eye recurrency of subretinal fluid at the time of presentation and its resolution under therapy. Uninvolved left eye shows normal foveolar depression

Under supportive therapy with brinzolamide 10 mg/ml eye drops 2x/d and eplerenone 50 mg/d fluid withdraw in two month’s time, visual acuity turned to 20/20 and subretinal fluid resolved in SD-OCT (Fig. 1). Our therapy was beyond guidelines for CSC treatment, but was adapted to the individual patient and to clinical experience.

#### Laboratory assessment at admission

No abnormalities in routine parameters apart from pre-diagnosed hypercholesterinemia. Leucocyte count (5400/µl at admission, 5300/µl at dismissal) and C-reactive protein values (1.1 mg/l each at admission and at dismissal) as potential markers of inflammation were in the normal range at the beginning and end of the psychotherapeutic treatment.

#### Laboratory assessment (week 10 of inpatient stay)

To assess the biological stress reaction, we analyzed several stress marker (Table 1).

**Table 1:** Analyzed stress markers

Parameter	Value	Unit	Reference	Interpretation
Cortisol	55	ng/ml	8–10 h: 50-120	Ok
Cortisone	13.4	ng/ml	4.8-19.5	Ok
DHEA-S	1003	ng/ml	889-4270	Ok
Testosterone	2.89	ng/ml	3.5-11.5	Reduced
Androstendione	0.41	ng/ml	0.3-3.1	Ok
17-Hydroxy-Progesterone	0.46	ng/ml	<1.6	Ok
21-Deoxycortisol	n.d.	ng/ml	healthy n.d	Ok
Corticosterone	1.65	ng/ml	0.1-2	Ok
11-Deoxycortisol	0.19	ng/ml	0.05-0.3	Ok
11-Deoxycorticosterone	n.d.	ng/ml	0.02-0.15	Ok
Progesterone	n.d.	ng/ml	healthy n.d.	Ok

DHEA-S, dehydroepiandrosterone sulfate; n.d., not detectable

Testosterone values were decreased in the patient. Other analyzed stress markers were within the reference values.

To get deeper insight into the biological stress reaction, we analyzed the day profile of cortisol secretion using a liquid chromatography-tandem mass spectrometry (LC-MS/MS) assay (Table 2).

**Table 2:** Cortisol day profile

Parameter	Time	Value	Unit	Reference	Interpretation
Cortisol profile	morning	1.10	ng/ml	0.2-3.59	Ok
	noon	3.12	ng/ml	0.2-2.42	Increased
	evening	1.07	ng/ml	0.2-1.24	Ok

While morning and evening values were in the normal range, the noon value showed an increased cortisol level. This peak was indicative of a stress reaction [10].

According to the CARE guidelines [11–13], a timeline is provided for an overview of the patient’s course of care (Table 3).

**Table 3:** Timeline

Week	Diagnostics / Treatment	Subjective stress reaction / CSC	Stress biomarkers
1	Diagnostic assessment,		
	Psychoeducation		
2	Psychoeducation, Stabilization		
3	Psychoeducation, Stabilization		
4	EFDR (two sessions)	Severe	
5	EFDR (one session), Stabilization	Severe	
6	EFDR (one session), Stabilization	Severe	
7	EFDR (one session), Stabilization	Severe	
8	EFDR (one session), Stabilization	Severe	
9	No psychotherapy	CSC	
10	Stabilization, Preparation of dismissal	CSC	Increased cortisol

CSC, central serous chorioretinopathy; EFDR, Eyes Fixated Desensitization and Reprocessing

### Diagnostic assessment

For psychiatric diagnostic assessments, we used clinician-rated self-reported questionnaires (ETI, BDI-II). We analyzed biological stress markers using a liquid chromatography-tandem mass spectrometry (LC-MS/MS) assay.

The patient was diagnosed with a post-traumatic stress disorder, a medium depressive episode in the course of a recurrent depressive disorder, psychological and behavioral factors in the context of arterial hypertension (exacerbations of arterial hypertension upon episodes of psychosocial stress), as well as with bruxism. Furthermore, he received the diagnosis of a central serous retinopathy, obesity grade I WHO, hypercholesterinemia, and bursitis of the right shoulder.

### Therapeutic intervention

The patient received 10 weeks of trauma-oriented inpatient psychotherapy at the Psychosomatic Division of the Ebermannstadt Hospital, Hospital Forchheim-Fränkische Schweiz, Ebermannstadt, Germany, which is a cooperation hospital of the Friedrich-Alexander University Erlangen-Nürnberg (FAU), Germany.

At admission, the therapeutic foci were on stabilizing methods: creation of an individual disorder model, psychoeducation, imagination exercises, enhancement of self-perception and self-care, and an overview of traumatic events and resources (lifeline, from Narrative Exposure Therapy (NET), 12,13).

Four weeks after admission and stabilizing treatment, the patient underwent trauma-focused psychotherapy with a modified version of Eye Movement Desensitization and Reprocessing (EMDR; 14): instead of following the moving hand of the therapist, we asked him to look at the fixated hand of the therapist (EFDR, Eyes Fixated Desensitization and Reprocessing;,15). We used this modification in order to prevent retinal detachment, as previous psychotrauma therapists had reported from their clinical experience of cases of retinal detachment upon classic EMDR in patients affected by eye diseases that can cause retinal detachment. Since CSC is a risk factor for retinal detachment, we chose EFDR for safety reasons. EFDR has proven non-inferior to EMDR regarding outcomes of PTSD symptomatology in a previous study by Sack et al. [15]. The patient preferred EFDR to other ways of bilateral stimulation, e.g. tactile stimulation. We assessed protocol adherence via video recording.

EMDR [14] is an evidence based treatment for PTSD [16] that consists of an activation of a traumatic memory with the most disturbing image, negative thought about oneself, emotion and bodily perception which is then reprocessed with phases of eye movements that the patient follows with his/her eyes (in our case: fixated hand which is looked at by the patient, Eyes Fixated Desensitization and Reprocessing EFDR). Usually, symptom distress reduces in the course of several sessions of EMDR therapy.

As the most disturbing memory, the patient chose the sexual abuse in his infancy. Upon EFDR treatment, he had severe bodily reactions such as trembling, high tension, exhaustion, pain in different parts of the body (e.g. headaches, pain in the back and the jaws) and exacerbation of bruxism. Furthermore, self-depreciating thoughts, shame and disgust occurred. Later, emotions like anger came up, which then converted into sadness. After initial deterioration of sleep, he reported an improvement of sleep quality and quantity in the course of the psychotherapeutic treatment. During this treatment, tension and bodily symptoms reduced and the patient reported that he perceived the sessions as very exhausting, but helpful. Apart from EFDR, he used weekly stabilization psychotherapy sessions and body and art therapy sessions to integrate the stressful memories that came up which the patient perceived mostly on the bodily level and to focus on resources and distancing techniques.

After six sessions of EFDR therapy, acute vision loss with blurred vision and image distortion of the right eye occurred. Thus, we presented the patient at the Department of Ophthalmology of Friedrich-Alexander University Erlangen-Nürnberg (FAU), Germany. Our colleagues confirmed an acute episode of the patient’s pre-known CSC of the right eye. Consequently, the colleagues prescribed brinzolamide eye drops and eplerenone.

In order to reduce stress, we stopped EFDR treatment and continued with stabilizing and self-compassion interventions and the activation of resources.

#### Medication at dismissal

Sertraline 150 mg 1-0-0, quetiapine retard 25 mg 0-0-1, lercanidipine 10 mg 1-0-1, *vitamine D 1000 IE 1-0-0, brinzolamide eye drops twice a day (affected eye), eplerenone 50 mg 1-0-0 (italics: new medication).*

### Follow-up and outcomes

One year after dismissal, the patient reported that depressive and post-traumatic symptoms including lack of motivation, tiredness, flashbacks, sleep problems and pain were still present. After dismissal, he continued outpatient (mainly online due to the pandemic) psychotherapeutic treatment with a focus on everyday problems and being present. About nine months after his dismissal, he managed to be put on the waiting list for a trauma-focused psychotherapy. Furthermore, he continued doing occupational therapy. Sertraline has been stopped and substituted by duloxetine. However, he reported that he did not perceive any benefit neither from sertraline nor from duloxetine. Furthermore, after dismissal, he received an augmentation of his quetiapine dose (from quetiapine retard 25 mg to quetiapine retard 300 mg 0-0-0-1, quetiapine 75 mg 0-0-0-1, and quetiapine on demand 25–50 mg), with no significant effect. He continued to take lercanidipine 10 mg 1-0-1-0 because of his arterial hypertension.

In psychometric diagnostics at a one-year follow-up examination, he exhibited a medium depressive episode with a BDI-II score of 47 points and the symptoms of a post-traumatic stress disorder with 55 points in the ETI, thus similar values as those at dismissal.

Regarding his CSC, the patient described that there had been a significant improvement in vision and that the “bubble” in his eye had disappeared again three months after dismissal. However, he reported that three-dimensional vision was still compromised. He had continued to take eplerenone 50 mg 1-0-0-0 for three months after dismissal, which he tolerated well. Later after dismissal, he was informed that his disability pension had been denied. This caused significant stress and led to another episode of CSC, which again had been treated by eplerenone.

## Discussion

To our knowledge, this is the first case report on a patient with comorbid PTSD and CSC undergoing trauma-confronting psychotherapy. During trauma-focused treatment for PTSD, a relapse of CSC occurred.

The comorbidity of PTSD and CSC in our patient is in line with previous research showing a strong association between PTSD and the development of CSC [6]. Our observation is in accordance with the assumption that psychological stress might be one of the most important risk factors for exacerbations of CSC [5]. We also found an increase in cortisol levels after CSC occurred, which is indicative of a stress reaction. In line with this, the use of steroids was earlier shown to be a risk factor for CSC [3]. PTSD was suggested to be associated with a blunted cortisol secretion [17]. However, it was shown that responders had increased cortisol levels after therapy compared with non-responders [18]. Thus, the specific stress situation upon successful trauma-confronting psychotherapy could result in changes in the diurnal cortisol profile and therefore mediate the risk for CSC.

The study of side effects of various psychotherapeutic treatments has long been neglected. Between 5% and 20% of all patients receiving psychotherapeutic treatment experience relevant side effects, with psychological stress and family or marital stress being the most commonly reported [19]. Other side effects described include symptom exacerbation, emergence of new symptoms, suicidality, impact on professional and social life, stigmatization and undermining of self-efficacy [20]. Nevertheless, psychological intervention studies usually do not report on potential side effects. For a few years now, efforts have been made to pay more attention to possible side effects [21]. When comparing different instruments for assessing negative effects of psychotherapy, it became apparent that although there is a large number of possible domains of adverse effects, there is disagreement about a precise definition and conceptualization of adverse effects and their respective relevance [22].

Since a core idea of medical treatment is ‘primum nil nocere’, i.e. not to harm, offering adequate psychotherapeutic treatment for PTSD with comorbid CSC is challenging. On the one hand, for recovery of PTSD, a trauma-specific psychotherapeutic approach including trauma-confronting elements is crucial. On the other hand, however, trauma-confrontation usually causes a temporary increase of stress symptoms, which then can cause exacerbations of the eye disease when a history of CSC is present. Since there is no evidence on long-term outcomes for both diseases in the course of a longer trauma-focusing treatment, we can only speculate what would be the best therapy options for patients presenting with both disorders.

First, we suggest continuous monitoring for CSC symptoms upon psychotherapeutic treatment, which can always cause stress due to the actualization of stress and problems. Thus, it seems reasonable to adjust the “dosage” of trauma-confronting psychotherapy in individuals affected by both disorders. At admission for psychotherapy, it might be beneficial to administer prophylactic doses of drugs that are usually given for acute exacerbations of CSC in order to prevent from CSC crises upon psychotherapy. It might furthermore be beneficial to screen CSC patients for PTSD, and to inform patients about potential exacerbations of the eye disease upon psychotherapy if patients present with PTSD and comorbid CSC.

A strength of our report is that the findings are partly novel and of clinical relevance. Furthermore, the case report includes several biological stress parameters, e.g. a diurnal cortisol profile. This is helpful to assess the underlying pathophysiology for this case. The findings are limited by a lack of pre-treatment assessment of the ophthalmologic situation and of stress biomarkers. Hence, it is difficult to discern if biomarkers have increased and if CSC has recurred solely due to the psychotherapy sessions or would have recurred anyway. Furthermore, we have not re-measured cortisol levels after the patient’s dismissal and thus have no information about cortisol levels accompanying the second exacerbation once the patient’s disability pension was denied. Another limitation is that we conducted clinical diagnosis and self-report measures instead of structured clinical interview diagnosis. Furthermore, we applied EFDR instead of EMDR including eye movements or other forms of bilateral stimulation due to the contraindication of eye movements in our case and due to patient choice.

## Conclusions

CSC may exacerbate upon psychotherapeutic, especially trauma-confronting, treatment. In the future, psychotherapists and ophthalmologists should collaborate and monitor patients presenting with comorbid PTSD and CSC. This includes assessing for trauma and PTSD in CSC, and, vice versa, assessing for chronic stress-related somatic conditions in PTSD before the onset of psychotherapeutic treatment. Consequently, our main conclusion is that psychotherapists should screen for conditions that might exacerbate upon stress and reactualization of traumatic memories in PTSD patients undergoing trauma-confronting psychotherapy.

## Patient perspective

We asked the patient to report on his experiences during and after the inpatient stay:

‘The inpatient stay has been quite beneficial to me. However, I always expect too much from myself; nothing has changed about that. The psychotherapeutic sessions have been good; but when many men joined the therapy group, my stress level was again extremely high, and I still do not feel comfortable among men. After the EFDR sessions, stress levels were very high; however, I believe it has helped me to ‘tidy up’ memories.

The stay has helped me to get calmer, even though this has been really challenging in the beginning. However, I am still restless and cannot ‘switch off’. Progressive muscle relaxation has not been relaxing to me at all, but the opposite: rest is my biggest enemy in myself.

Depressive symptoms have slightly reduced, but I wouldn’t call it the life of a ‘normal’ person. If my shoulder is associated with my mental state I do not know; and the eye is the most important to me, ‘cause every day I am afraid that it will get worse again. Sleeping hasn’t been working at all until today, ‘cause I still wake up often even though I’m taking medication and then I begin to ruminate; I force myself to fall asleep, respectively.’

## Data Availability

All data generated or analysed during this case study are included in this published article.

## Abbreviations

CSC: Central Serous Chorioretinopathy
PTSD: Post-Traumatic Stress Disorder
SD-OCT: spectral-domain optical coherence tomography
EMDR: Eye Movement Desensitization and Reprocessing
EFDR: Eyes Fixated Desensitization and Reprocessing
BDI-II: Beck Depression Inventory II
ETI: Essen Trauma Inventory
